# Thrombelastography Compared with Multiple Impedance Aggregometry to Assess High On-Clopidogrel Reactivity in Patients with Atrial Fibrillation Undergoing Percutaneous Coronary Intervention

**DOI:** 10.3390/jcm11144237

**Published:** 2022-07-21

**Authors:** Diona Gjermeni, Hannah Vetter, Sofia Szabó, Viktoria Anfang, Stefan Leggewie, David Hesselbarth, Daniel Duerschmied, Dietmar Trenk, Christoph B. Olivier

**Affiliations:** 1 Department of Cardiology and Angiology, Heart Center Freiburg University, Faculty of Medicine, University of Freiburg, 79106 Freiburg, Germany; diona.gjermeni@uniklinik-freiburg.de (D.G.); hannah.vetter@uniklinik-freiburg.de (H.V.); sofia.szabo@uniklinik-freiburg.de (S.S.); viktoria.anfang@uniklinik-freiburg.de (V.A.); stefan.leggewie@uniklinik-freiburg.de (S.L.); david.hesselbarth@uniklinik-freiburg.de (D.H.); dietmar.trenk@uniklinik-freiburg.de (D.T.); 2 Department of Cardiology, Angiology, Haemostaseology and Medical Intensive Care, University Medical Center Mannheim, Medical Faculty Mannheim, Heidelberg University, 68167 Mannheim, Germany; daniel.duerschmied@uniklinik-freiburg.de; 3 European Center for AngioScience (ECAS) and German Center for Cardiovascular Research (DZHK) Partner Site Heidelberg/Mannheim, 68167 Mannheim, Germany

**Keywords:** atrial fibrillation, percutaneous coronary intervention, platelet reactivity, thrombelastography, multiple electrode aggregometry

## Abstract

Background: High on-clopidogrel platelet reactivity (HPR) following percutaneous coronary intervention (PCI) is associated with increased ischemic risk. It is unclear whether conventional definitions of HPR apply to patients with concomitant oral anticoagulation (OAC). This study aimed to compare the performance of multiple platelet aggregometry (MEA) and thrombelastography (TEG) to detect HPR in patients with atrial fibrillation (AF) and indication for an OAC. Methods: In this observational single-center cohort study, MEA and TEG were performed in patients with AF with an indication for OAC on day 1 to 3 after PCI. The primary outcome was HPR as assessed by MEA (ADP area under the curve ≥ 46 units [U]) or TEG (MA_ADP_ ≥ 47 mm), respectively. The secondary exploratory outcomes were a composite of all-cause death, myocardial infarction (MI) or stroke and bleeding, as defined by the International Society on Thrombosis and Hemostasis, at 6 months. Results: Platelet function of 39 patients was analyzed. The median age was 78 (interquartile range [IQR] was 72–82) years. 25 (64%) patients were male, and 19 (49%) presented with acute coronary syndrome. All patients received acetylsalicylic acid and clopidogrel prior to PCI. Median (IQR) ADP-induced aggregation, MA_ADP_, TRAP-induced aggregation, and MA_thrombin_ were 9 (6–15) U, 50 (43–60) mm, 54 (35–77) U and 65 (60–67) mm, respectively. The rate of HPR was significantly higher if assessed by TEG compared with MEA (25 [64%] vs. 1 [3%]; *p* < 0.001). Within 6 months, four (10%) deaths, one (3%) MI and nine (23%) bleeding events occurred. Conclusion: In patients with AF undergoing PCI, the rates of HPR detected by TEG were significantly higher compared with MEA. Conventional cut-off values for HPR as proposed by consensus documents may need to be re-evaluated for this population at high ischemic and bleeding risk. Further studies are needed to assess the association with outcomes.

## 1. Introduction

Approximately one-third of patients with atrial fibrillation (AF) have coexisting coronary artery disease often demanding percutaneous coronary intervention (PCI) [1,2]. Guidelines recommend clopidogrel and a direct oral anticoagulant (DOAC) [3,4,5,6] for most patients with AF undergoing PCI [1,7,8]. The omittance of acetylsalicylic acid (ASA) reduces bleeding risk [3]. Still, 6–8% of AF patients experience ischemic events within 6 months after PCI [1,7]. In patients treated with dual antiplatelet therapy (DAPT), high inter-individual variability of response to clopidogrel therapy has been described. High on-clopidogrel platelet reactivity (HPR) was reported in 21% [9] to 39% [10] of patients depending on the assay and patient characteristics. HPR is associated with increased risk for ischemic events [11,12,13]. Guidelines and consensus documents recommend that platelet functions testing (PFT) could be considered for patients as a tool to adapt antiplatelet therapy [7,13]. Viscoelastic tests such as thrombelastography (TEG) have been widely used as tools to assess hemostasis in patients undergoing surgery, obstetrics and trauma patients, but the use in cardiology and the experience in patients treated with oral anticoagulation is limited.

The association of HPR with ischemic risk might be more pronounced in patients with concomitant DOAC, since ASA is often omitted [1]. It is unclear whether conventional definitions of HPR apply to patients with concomitant oral anticoagulation (OAC) and which PFT is most suitable for detecting HPR for risk stratification in these patients.

This study aimed to compare the performance of multiple electrode aggregometry (MEA) and TEG to detect HPR in patients on clopidogrel and OAC.

## 2. Methods

### 2.1. Study Design and Clinical Characteristics

Patients were enrolled in an observational prospective single-center cohort study between May 2020 and May 2021. The ethics committee of the University of Freiburg, Germany, approved the protocol and amendments (registry number 194/20). All patients provided written informed consent to participate in the study. MEA and TEG measurements were performed in 39 consecutive patients at the Department of Cardiology and Angiology I at Heart Center Freiburg University. All patients underwent coronary stent implantation and were treated with ASA periprocedurally. Patients were eligible if 18 years or older, had AF with an indication for OAC (CHA_2_DS_2_VASC-score ≥ 1 for males, ≥2 for females) and received PCI within the last 3 days. Exclusion criteria were history of stent thrombosis, a platelet count below 50 × 10^3^ platelets per μL blood, therapy with GPIIb/IIIa-inhibitors in the last 24 h and use of prasugrel or ticagrelor in the last 7 days.

### 2.2. Blood Samples

Venous blood samples were taken using 21 G butterfly needle (Safety-Multifly^®^-Set, Sarstedt, Nümbrecht, Germany) to a final concentration of >15 μg/mL r-hirudin (SARSTEDT Monovetten, Nümbrecht, Germany) for MEA and 17 IU/mL Li-heparin (Becton, Dickinson and Company, Heidelberg, Germany) for TEG. Blood samples were stored at room temperature, and platelet function was analyzed according to the manufacturer’s instructions.

### 2.3. Multiple Electrode Aggregometry

Multiple electrode aggregometry (MEA, Roche Diagnostics, Risch-Rotkreuz, Switzerland) was performed on day 1 to 3 after PCI. Whole blood was stimulated with adenosine diphosphate (ADP; final concentration 6.4 μM) or thrombin receptor activating peptide-6 (TRAP; final concentration 32 μM), respectively. HPR_MEA_ was defined as ADP area under the curve [AUC] ≥ 46 U according to the expert consensus on platelet function and genetic testing [13]. For TRAP-induced platelet aggregation, reference values per manufacturer were 94–156 U [13].

### 2.4. Thrombelastography

Thrombelastography was performed with TEG 6s Hemostasis Analyzer (Haemonetics Corp., Boston, MA, USA). A multichannel cartridge holding dried reagents was used. Approximately 400 μL blood were automatically aspirated into the testing area and mixed with the required reagents. Then, 2 μM ADP was used as a reagent for platelet function, and kaolin with heparinase (concentration > 1800 IU/mL), eliminating the effects of heparin, was used for overall aggregability. The blood was automatically exposed to ultrasound pulses (20–500 Hz frequency), changing during coagulation depending on clot strength. The maximum amplitude (MA, [mm]) was determined to describe the maximum clot strength. HPR_TEG_ was defined as a MA_ADP_ ≥ 47 mm after stimulation with ADP. Kaolin-activated channel HKH-channel was used for MA_Thrombin_ (reference values per manufacturer: 53–68 mm).

### 2.5. Study Outcomes and Follow-Up

The primary outcome was the rate of HPR as assessed by TEG and MEA in patients with AF undergoing PCI. The exploratory secondary outcomes were a composite outcome of all-cause death, myocardial infarction (MI) or stroke [14] and bleeding (major or non-major clinically relevant [NMCR]) as defined by the International Society on Thrombosis and Haemostasis (ISTH) [15] at 6 months. Follow-up was assessed by a structured telephone interview at 6 months ±2 weeks after inclusion in the study. Clinical outcome events were adjudicated by two independent physician reviewers blinded to MEA and TEG results. Major discrepancies were resolved by the PI (CBO) who was blinded to MEA and TEG results.

### 2.6. Statistical Considerations

Data are presented as number with percentage for binomial variables and median with interquartile range for continuous variables. Categorical variables were compared using Fisher’s exact test. Continuous variables were compared with a 2-sided unpaired *t*-test or Mann–Whitney U test. Pearson’s correlation test was performed to evaluate the correlation between TEG and MEA. A *p* value of 0.05 or less was considered significant. Data were analyzed with Prism 12.0.13 (GraphPad Software, La Jolla, CA, USA) and SPSS 27.0.0.1 (SPSS Inc., Chicago, IL, USA).

## 3. Results

### 3.1. Patient Population and Medication

Thirty-nine patients were enrolled between day 1 and 3 after PCI. Clinical baseline characteristics are shown in Table 1. The median age was 78 (72–82) years and 25 (64%) were male. Median CHA_2_DS_2_-VASC score was 5 (4–6) and median HAS-BLED 3 (3–4). Arterial hypertension was reported in 38 (97%) patients, hyperlipidemia in 30 (77%) patients, diabetes mellitus in 15 (38%) patients and family history of coronary artery disease (CAD) in 10 (26%) patients. Median platelet count was 210 (166–297) × 10^3^/μL. Nineteen (49%) patients underwent PCI due to acute coronary syndrome (ACS), 8 (21%) patients had a history of stroke/transient ischemic attack (TIA), and 8 (21%) patients had a history of gastrointestinal or intracranial bleeding.

The periprocedural antithrombotic therapy and the antithrombotic therapy at discharge are shown in Table 2. Thirty-four (87%) patients received 250 or 300 mg periprocedural ASA, and five (13%) were on 100 mg ASA maintenance therapy. Thirty-six (92%) patients received an initial, periprocedural dose of 300 or 600 mg clopidogrel. Three (8%) patients had 75 mg clopidogrel per day as maintenance therapy. Oral anticoagulation was interrupted before PCI in six (15%) patients. Low-molecular-weight heparin was used in four (10%) patients.

At discharge, all patients were prescribed clopidogrel. ASA was discontinued at discharge in 29 (74%) of the patients. OAC therapy at discharge consisted in the vast majority of patients of DOACs: 8 (20%) edoxaban, 10 (26%) apixaban, 16 (41%) rivaroxaban, and 3 (8%) patients received a vitamin K antagonist (VKA). Two (5%) of the patients had no OAC at discharge.

### 3.2. Platelet Aggregation Assessed by MEA and TEG

**Figure 1:**
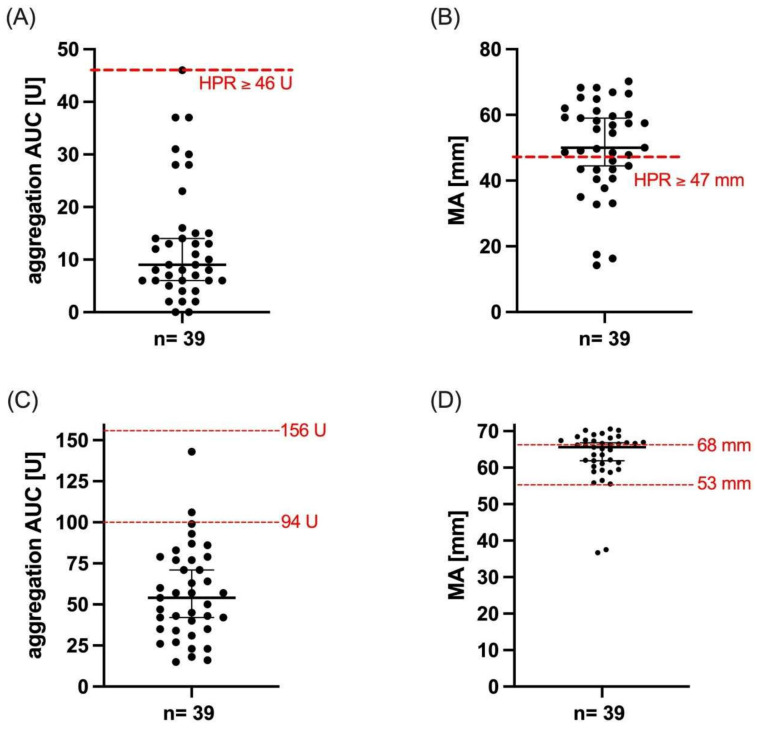
**Platelet reactivity of patients with atrial fibrillation undergoing percutaneous intervention.** (**A**) ADP- and (**C**) TRAP-induced aggregation as assessed by MEA and MA_ADP_ (**B**) and MA_Thrombin_ (**D**) as assessed by TEG. Median and interquartile range are represented by black lines. Red lines indicate conventional cut-off values (ADP AUC ≥ 46 U and MA_ADP_ ≥ 47 mm) or reference values suggested by the manufacturer (TRAP AUC 94-156 U, MA_Thrombin_ 53–68 mm), respectively.

Median ADP-induced aggregation AUC was 9 (IQR 6–15) U and median MA_ADP_ was 50 (IQR 43–60) mm (Figure 1).

**Figure 2:**
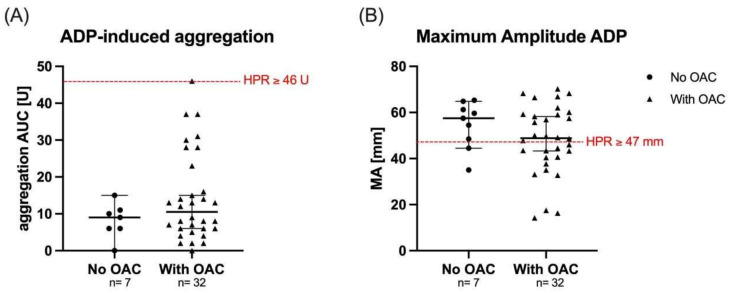
**Platelet reactivity according to oral anticoagulation.** Aggregation values for ADP-induced aggregation as assessed by MEA (**A**) and MA_ADP_ as assessed by TEG (**B**) with and without OAC therapy. Red line represents HPR cut-off and black line median with interquartile range.

The rate of HPR was significantly higher if assessed by TEG compared with MEA 25 (64%) vs. 1 (3%); *p* < 0.001. Thirty-two (82%) patients received OAC on the day of platelet functioning measurement. No significant difference in ADP-induced aggregation (9 U vs. 11 U; *p* = 0.09) and MA_ADP_ values (59.6 mm vs. 48.5 mm, *p* = 0.06) were shown when OAC was discontinued at measurement time compared with measurements performed under OAC therapy (Figure 2).

Median TRAP-induced aggregation was 54 (IQR 35–77) U and MA_Thrombin_ was 66 (IQR 60–67) mm.

**Figure 3:**
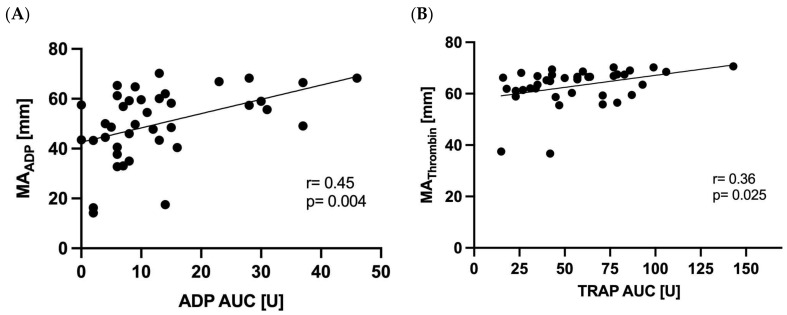
**Correlation of multiple electrode aggregometry with thrombelastography.** ADP AUC with MA_ADP_ (**A**) and TRAP AUC with MA_Thrombin_ (**B**).

ADP-induced aggregation correlated with MA_ADP_ (r = 0.45, *p* < 0.004). TRAP-induced aggregation correlated significantly with MA_Thrombin_ (r = 0.36, *p* = 0.025, Figure 3).

### 3.3. Exploratory Clinical Outcomes

The composite exploratory ischemic outcome of death, MI, or stroke occurred in five (13%) patients (five [10%] deaths and one [3%] MI; Table 3). The secondary exploratory outcome NMCR or major bleedings occurred in nine (23%) patients (three [8%] NMCR and six [16%] major bleedings). No significant association of HPR status was observed as assessed by both TEG and MEA with exploratory outcomes. The study had not been powered to detect an association with clinical outcomes.

## 4. Discussion

The main finding of this study is that in patients with AF with an indication for OAC undergoing PCI, the rate of HPR detected by TEG was substantially higher when compared with MEA.

### 4.1. Multiple Electrode Aggregometry

In patients treated with DAPT, MEA could be considered to assess platelet function and guide antithrombotic therapy [10,13]. The prevalence of HPR as assessed by MEA in patients with DAPT following PCI ranged from 15% up to 39% in patients treated with DAPT after PCI [10,11]. The rate of HPR as assessed by MEA in the present study was low (3%). This might be attributed to the higher rate of AF in the present cohort compared with other studies. AF prevalence increases with age and the presence of comorbidities associated with decreasing platelet reactivity [13]. In a previous study, in patients with AF undergoing PCI, the rate of HPR as assessed by MEA was 15% [11]. However, the majority of these patients were treated with VKA. Studies suggest that VKA attenuates the efficacy of clopidogrel and increases HPR rates [12,16]. In another study in patients with clopidogrel and the DOAC dabigatran, median ADP-induced aggregation was 326 [268–462] (corresponding to 33 [27–46] U as used in the present study) and similar to patients receiving a VKA [17]. Smaller trials investigated platelet aggregation with different assays in patients treated with clopidogrel, ASA and DOAC therapy dabigatran [17,18], edoxaban [19] and low dose rivaroxaban [20]. No effect on clopidogrel-mediated platelet inhibition from different DOACs was observed when platelet aggregation was assessed by MEA or light transmission aggregometry [17,18,19,20]. However, DOAC therapy had been initiated recently in these patients, and it has been stated that alterations in platelet functions may be present when therapy with DOAC is used for a longer period [21]. This might be due to a change in the expression profile of thrombin receptor [21]. Overall, 78% of patients included in the present study were pre-treated with DOAC and not with a VKA. Another study showed that after the first intake of rivaroxaban and dabigatran, ADP-induced aggregation in patients taking clopidogrel was not affected [22]. In the present study, the majority of patients had TRAP-induced aggregation below the reference values. This indicates that the reduced platelet reactivity in these patients might not be exclusively ADP pathway dependent.

### 4.2. Thrombelastography

Gurbel et al., showed a 10-fold increase in ischemic risk in patients after stenting treated with DAPT when HPR was defined as MA_ADP_ ≥47 mm as assessed by TEG [23]. Studies reported HPR rates varying from 21% to nearly 50% in patients undergoing stent implantation after receiving loading with clopidogrel [9,24]. Compared with these rates, in the present study, the rate of HPR assessed by TEG seems high (64%). The patient cohort was elderly and presented with a significant thromboembolic risk. TEG measurements varied significantly between middle-aged and elderly healthy men and women [25]. Others reported a high intra-assay variability of TEG when comparing healthy donors with patients with ASA therapy [26]. These data indicate that reference values might vary according to age.

Smaller trials have evaluated TEG performance in patients treated with triple antithrombotic therapy. Edoxaban prolonged in a dose-dependent manner the speed of thrombin formation when assessed by TEG [19]. Other DOAC such as dabigatran were compared with placebo in patients with coronary artery disease without an indication for oral anticoagulation. Dabigatran affected the parameters related to thrombin formation with no effect on the kaolin-activated clot strength. MA_ADP_ was significantly higher in the group treated with dabigatran, showing consistent results with the high median MA_ADP_ reported in this study [18].

### 4.3. Performance of Multiple Electrode Aggregometry and Thrombelastography

In the present study, we identified a significant correlation of TRAP-induced aggregation with MA_Thrombin_ as well as ADP-induced aggregation with MA_ADP_. One study performed on 10 healthy volunteers also suggested that linear models could be generated between TEG and MEA [27], but no other studies have compared the performance of TEG with MEA in patients undergoing PCI.

Despite this correlation, the rates of HPR were substantially different between MEA and TEG. This difference might be explained by (1) pre-analytic factors, such as anticoagulant in the blood sampling tube and/or (2) the test principles. Thrombelastography quantifies clot formation, clot strength, and clot stability. MEA principle relies on platelet aggregation and might depend more on isolated platelet function compared with TEG. Consistent with this hypothesis, TEG is less affected by platelet count compared with MEA [28]. TEG, different from other assays, does not bypass other pathways that lead to platelet activation [29].

Most studies evaluating methods that measure platelet aggregation were performed in a setting of only DAPT therapy, have small sample sizes and were not randomized, explaining the conflicting results about the performance of PFT in different patient cohorts.

Further clinical trials are necessary to better explain the mechanisms of these findings.

### 4.4. Exploratory Outcomes

Death, MI, or stroke occurred in five (13%) patients. Secondary exploratory outcome consisting of NMCR or major bleedings occurred in nine (23%) patients. Different randomized trials compared triple antithrombotic therapy (TAT) with DOAC vs. VKA in patients with AF undergoing PCI and reported lower incidence of ischemic and bleeding events [3,4,5,6].

Chance and the reduced robustness due to the small size of the here presented study might explain the high event rate.

This study was not powered to evaluate the association of HPR with the secondary exploratory outcomes. Further studies need to determine and validate cut-off values to identify patients at risk of ischemic events.

### 4.5. Study Limitations

The different HPR rates between both methods were observed during exploratory measurements and were not part of a primary study hypothesis. Thus, this current analysis should be considered hypothesis generating. Further studies are necessary to compare these methods and to investigate potential mechanism to explain the observed differences in the assays. Since these findings were part of exploratory analyses, no sample size calculation had been performed regarding the performance of the assays. The low number of events indicates that the study is underpowered to assess association of HPR with ischemic and bleeding outcomes.

## 5. Conclusions

In patients with AF undergoing PCI, rates of HPR detected by TEG were significantly higher as compared with MEA. These differences indicate that conventional cut-off values for HPR as proposed by consensus documents may need to be re-evaluated for this population at high ischemic and bleeding risk. Further studies will need to investigate the mechanisms of this observation and determine the association of HPR with outcomes according to different definitions.

## Figures and Tables

**Table 1:** Clinical baseline characteristics.

Baseline Characteristic	All *n* = 39	
Demographics		
Age, years	78	(72–82)
Male	25	(64%)
BMI, kg/m2	27	(24–31)
Medical History		
Type of atrial fibrillation		
Paroxysmal	24	(61%)
Persistent	10	(26%)
Permanent	5	(13%)
CHA2DS2–VASc score	5	(4–6)
HAS–BLED score	3	(3–4)
Hypertension	38	(98%)
Diabetes	15	(39%)
Hyperlipidemia	30	(77%)
Renal impairment	17	(44%)
Nicotine abuse	10	(26%)
GI–/intracranial bleeding	8	(21%)
Prior stroke/TIA	8	(21%)
Prior PCI	10	(26%)
Family history of CAD	10	(26%)
Indication for PCI		
ACS	19	(49%)
Elective	20	(51%)
Thrombocyte count, D7103/μL	210	(166–297)
IPF in %	3	(3–5)

Values are *n* (%) or median (interquartile range). Abbreviations: ACS, acute coronary syndrome; BMI, body mass index; CAD, coronary artery disease; GI–bleeding, gastrointestinal bleeding; IPF, immature platelet fraction; PCI, percutaneous coronary intervention; TIA, transient ischemic attack.

**Table 2:** Periprocedural medication and medication at discharge.

Medication	All *n* = 39	
ASA		
250 mg loading	14	(51%)
300 mg loading	20	(51%)
Maintenance therapy	5	(13%)
Clopidogrel		
300 mg loading	12	(30%)
600 mg loading	24	(62%)
Maintenance therapy	3	(8%)
Bridging therapy	4	(11%)
OAC at time of measurement	32	(82%)
OAC at discharge		
Vitamin K antagonist	3	(8%)
Edoxaban	8	(21%)
Apixaban	10	(26%)
Rivaroxaban	16	(41%)

Values are *n* (%). Abbreviations: ASA, acetylsalicylic acid; OAC, oral anticoagulation.

**Table 3:** Exploratory clinical outcomes at 6 months ± 2 weeks according to the presence of HPR and no HPR as assessed by MEA and TEG.

			MEA				TEG			
	Total *n* = 39		HPR *n* = 1		No HPR *n* = 38		HPR *n* = 25		No HPR *n* = 14	
Death, MI, or stroke	5	(13%)	0	(0%)	5	(13%)	3	(12%)	2	(14%)
Death	4	(11%)	0	(0%)	4	(11%)	3	(12%)	1	(7%)
MI	1	(3%)	0	(0%)	1	(3%)	0	(0%)	1	(7%)
Stroke	0	(0%)	0	(0%)	0	(0%)	0	(0%)	0	(0%)
Bleeding events	23	(61%)	0	(0%)	23	(61%)	14	(56%)	9	(64%)
NMCR + major bleeding	9	(23%)	0	(0%)	9	(23%)	7	(28%)	2	(14%)
Major bleeding	6	(16%)	0	(0%)	6	(16%)	4	(16%)	2	(14%)
NMCR bleeding	3	(8%)	0	(0%)	3	(8%)	3	(12%)	0	(0%)
Minor bleeding	14	(37%)	0	(0%)	14	(37%)	7	(28%)	7	(50%)

Values are *n* (%). Abbreviations: HPR, high platelet reactivity; MI, myocardial infarction; NMCR, non-major clinically relevant bleeding.

## Data Availability

Data is not publicly available but can be available on reasonable request.
